# Arsenic Exposure and Cognitive Performance in Mexican Schoolchildren

**DOI:** 10.1289/ehp.9961

**Published:** 2007-05-22

**Authors:** Jorge L. Rosado, Dolores Ronquillo, Katarzyna Kordas, Olga Rojas, Javier Alatorre, Patricia Lopez, Gonzalo Garcia-Vargas, María del Carmen Caamaño, Mariano E. Cebrián, Rebecca J. Stoltzfus

**Affiliations:** 1 School of Natural Sciences, Universidad Autónoma de Querétaro, Division of Nutritional Sciences, Querétaro, Mexico; 2 Division of Nutritional Science, Cornell University, Ithaca, New York, USA; 3 Department of Psychology, Universidad Nacional Autónoma de México, Mexico City, Mexico; 4 Instituto Nacional de Ciencias Médicas y Nutrición, Mexico City, Mexico; 5 Medical School, Universidad Juárez del Estado de Durango, Gomez Palacio, Mexico; 6 Centro de Investigación y Estudios Avanzados, National Polytechnic Institute, Mexico City, Mexico

**Keywords:** arsenic, children, chronic exposition, cognitive performance, Mexico

## Abstract

**Background:**

Previous studies have suggested an effect of high arsenic concentration on cognitive and neurobehavioral function in humans.

**Objective:**

Our goal was to identify demographic and nutritional factors that are associated with As exposure and the influence of this exposure on cognitive function in school-age children.

**Methods:**

We recruited 602 children 6–8 years of age living within 3.5 km of a metallurgic smelter complex in the city of Torreón, Mexico, to participate in a cross-sectional evaluation. Of these, 591 had complete anthropometry, iron, and zinc status by biochemical measurements in serum, blood lead concentration (PbB), and arsenic in urine (UAs), and 557 completed several cognitive performance tests.

**Results:**

The mean for UAs was 58.1 ± 33.2 μg/L; 52% of the children had UAs concentrations > 50 μg/L, and 50.7% of children had PbB ≥10 μg/dL. UAs concentration was associated with low socioeconomic status. Nutritional status indicators were not related to UAs concentrations. Linear and logistic regressions adjusted for hemoglobin concentration, PbB, and sociodemographic confounders showed a significant inverse association between UAs and Visual–Spatial Abilities with Figure Design, the Peabody Picture Vocabulary Test, the WISC-RM Digit Span subscale, Visual Search, and Letter Sequencing Tests (*p* < 0.05). Boys excreted significantly more UAs (*p* < 0.05) and were affected on different cognitive areas than girls.

**Conclusions:**

Children living in an area contaminated with both As and lead showed that As contamination can affect children’s cognitive development, independent of any effect of lead.

Recent studies indicate that in several regions of the world, arsenic concentration in water is much higher than accepted levels (Smedley and Kinniburgh 2002). The concern with As contamination of drinking water is that consumption and use of this water in cooking can increase As exposure in humans (Del Razo et al. 2002). In Mexico, the amount of this contaminant in ground water varies from 10 to 5,000 μg/L (Del Razo et al. 1990). Del Razo et al. (1990; 1994) reported on the problem of As-contaminated groundwater in the Lagunera Region of northern Mexico, with > 50% of samples having As concentrations > 50 μg/L, which was the former level of reference set by the World Health Organization (International Programme on Chemical Safety 2001). The predominant type of As in 90% of the samples was pentavalent arsenic.

In 1977, the presence of As in potable water was reported in the city of Torreón, the main city in the Region Lagunera and the site of the most important metallurgic complex of Mexico. As concentration in Torreón’s water was up to 4–6 mg/L, far above the present 10-μg/L limit (Cebrian et al. 1994; Mandal and Suzuki 2002). Benin et al. (1999) evaluated heavy metal contamination of soil in three residential areas that surround the smelter and found that As levels had a median of 113 μg/g, and ranged from 78 to 287 μg/g. These levels exceeded the level at which the U.S. Environmental Protection Agency (EPA) designated cleanup goals for Superfund sites, 5–65 μg/g (U.S. EPA 1997).

Approximately 60–90% of the soluble inorganic arsenic (InAs) components are absorbed through the gastrointestinal tract (Hall 2002). In humans, InAs metabolism involves at least five metabolites that can exert toxic effects (Valenzuela et al. 2005). The measure of urinary As (UAs) excretion is a good biomarker for chronic exposure via drinking water [Agency for Toxic Substances and Disease Registry (ATSDR) 2000]. As concentration in urine, normally < 10 μg/L, can reach as high as 50 μg/L in adults and children living close to metal foundries (Carrizales et al. 2006; Polissar et al. 1990).

The negative consequences of As exposure in humans include respiratory, gastrointestinal, hematologic, hepatic, renal, dermic, neurologic, and immunologic effects (ASTDR 2000; Garcia-Vargas and Hernandez-Zavala 1996; International Programme on Chemical Safety 2001), many of which continue even after the contaminant source is controlled (Diaz-Barriga et al. 1997). As can also have detrimental effects on the central nervous system and cognitive development in children (International Programme on Chemical Safety 2001). Acute As exposure affects sensory nerves as well as the long axon neurons, which results in the clinical manifestation of numb extremities. Neurologic tests have shown nerve axonophathy and demyelination (Franzblau and Lilis 1989; Rodriguez et al. 2003; Yung 1984). As also affects the content of brain monoamines, and the concentrations of dopamine and serotonin in the hippocampus, hypothalamus, cerebral cortex, and the striatum (Itoh et al. 1990; Mejía et al. 1997; Rodríguez et al. 2003).

Few reports have suggested a detrimental effect of As exposure on cognitive development and function, including disturbed visual perception, problems with visuomotor integration, psychomotor speed, attention, speech, and memory. (Calderón et al. 2001; Rodriguez et al. 2003; Tsai et al. 2003). In this regard, the effects of As are similar to and could be confounded with the effects of other environmental contaminants such as lead. In fact, in many regions of the world, As exposure co-occurs with exposure to other contaminants such as lead (Carrizales et al. 2006; Diawara et al. 2006). Poorer performance on a range of cognitive tests has been reported in children with low to moderate lead exposure in Torreón (Kordas et al. 2004) and other settings (Canfield et al. 2003; De Burbure 2006; Lanphear et al. 2000).

In this study we identified demographic and nutritional factors that are associated with UAs concentration in school-age children. We also investigated the influence of As exposure on cognitive function in these children.

## Methods

### Subjects and design of the study

The study sample consisted of 602 children 6–8 years of age who attended first grade in nine public elementary schools located within 3.5 km of a metallurgic smelter complex in the city of Torreón, Mexico. Parents of all study subjects gave informed written consent before being enrolled. This study area was selected because the foundry contributes with toxic substances that affect a large proportion of the population. The effects of lead exposure in this population have been described previously (Dorea 2004; Flora 2002; Kordas et al. 2004, 2006; Rico et al. 2006; Rosado et al. 2006). Each child was examined to obtain anthropometry, nutritional status by biochemical measurements in serum, levels of blood lead (PbB) and UAs, and cognitive performance. Complete blood and urine samples were available for 591 children. The study was approved by the Human Subjects Research Committee at the Johns Hopkins Bloomberg School of Public Health and the Institute of Medical Sciences and Nutrition in Mexico.

### Anthropometry

Measurements were carried out according to standard methods (Habicht 1974). A trained person took all height and weight measurements. Weight was measured to the nearest 10 g using an upright scale (Model Express Plus, Torino, Mexico City, Mexico) before lunchtime to avoid variations due to immediate food ingestion. Height was measured with a 3-m standardized measurement board to the nearest 1 mm. All participants were measured without shoes and wearing only their school uniform without any sweater or jacket.

### Biochemical measures

A venous blood sample was collected from each child at the school, after an overnight fast. After blood sample collection, children were given a snack and a juice box. Blood was collected in 5-mL sodium heparin vacutainer trace-metal free tubes (Becton Dickinson, Franklin Lakes, NJ, USA). Hemoglobin (Hb) was analyzed at the school with a HemoCue Photometer (HemoCue Inc., Mission Viejo, CA, USA). Samples were transported in a cooler to be processed in the laboratory on the same day. Serum was obtained and aliquots stored at −80°C until analysis was done. We analyzed serum ferritin using an immunoradiographic method (Coat-A-count Ferritin IRMA). We analyzed zinc and copper concentration with an atomic absorption spectrophotometer (PerkinElmer Analyst 700, PerkinElmer, Norwalk, CT, USA). PbB and UAs were analyzed at the Center for Research and Advanced Studies in the National Polytechnic Institute in Mexico. This laboratory participates in two quality control programs, the Trace Elements External Quality Assessment Scheme at University of Surrey, United Kingdom, and the Interlaboratory Program of Quality Control at Zaragoza, Spain. For PbB measurement, we analyzed samples by duplicate using atomic absorption spectrophotometry (Zeeman 5100; PerkinElmer, Norwalk, CT, USA) (Miller et al. 1987), and those with a CV > 5% were reanalyzed. Lead in bovine blood (standard reference material 955b; National Institute of Standards and Technology, Gaithersburg, MD, USA) was used as the standard reference. For As measurement, a urine sample was collected in the morning after subjects had fasted overnight. It was collected in a plastic container with 100-mL capacity. Samples were transported with ice to the laboratory and a 25-mL aliquot was frozen at −20°C until analysis. On the day of analysis samples were unfrozen and warmed to 37°C in a container with boiling water; 2.5-mL aliquots with urine were placed in glass containers and then 2.5 mL HCl 2 M (6% w/v) was added. Samples were covered with clock glass and heated during 5 hr to 80°C. Then they were cooled to room temperature and transferred to a volumetric flask of 5 mL using HCl 2 M for dilution (Del Razo et al. 1999). The analysis was done with an atomic absorption spectrophotometer (PerkinElmer 3100; PerkinElmer), according to the procedure reported by Crecelius et al. (1986). UAs analysis included InAs, monomethylarsenic (MMAs), and dimethylarsenic (DMAs) and the sum of all metabolic species of arsenic. Zinc proto-porphirin (ZPP) was measured in whole blood with ZP Hematofluorometer (AVIV Biomedical, Lakewood, NJ, USA).

Zinc deficiency was considered when serum zinc was ≥65 mg/dL, anemia when Hb was < 12.4 g/dL, and ferritin deficiency ≤12 μg/L and copper deficiency when serum copper was < 80 μg/L. Elevated ZPP was considered when ≥ 70 μmol ZP/mol heme, high As concentration when As in urinary samples was > 50 μg/L, and PbB concentration was considered high when > 10 μg/dL.

### Cognitive measures

Cognitive evaluations included tests of memory, attention, problem solving, and vocabulary processes. Each participant required two working sessions to answer 14 pen-and-paper or computer touch-screen tests covering the various aspects of cognitive functioning. All children had previous experience with computers. The first session consisted of the Coding, Digit Span, and Arithmetic sub-tests of the Weschsler Intelligence Scale for Children Revised Mexican Version (WISC-RM) (Wechsler 1974, 1981), a test of number and letter sequencing (Reitan and Wolfson 1992), and the Cognitive Abilities Test (a computer-based test with four tasks: Stimulus discrimination, Sternberg memory, Visual Memory Span, and Visual Search) (Detterman 1988). These tests were all applied in this order on day 1. Day 2 consisted of a curriculum-based Math Achievement Test (MAT), a test of Visual–Spatial Abilities with Figure Design, and the Peabody Picture Vocabulary Test (PPVT-Spanish Edition) (Dunn et al. 1986), applied in this order. The WISC-RM and the PPVT were validated with Mexican-American populations; the rest of the tasks were piloted among 1st and 2nd graders in a public elementary school in Mexico City before the project began. Each of these tests has been described in detail in a previous publication showing the effects of PbB concentrations on cognitive performance of these children (Kordas et al. 2004, 2006; Rico et al. 2006).

### Demographics and socioeconomic status

A questionnaire was given to parents or caregivers of all children to identify the sociodemographic characteristics of the families. The questionnaire included questions to determine crowding, housing conditions, family possessions, and parents’ education level. These characteristics, except parents’ education level, were used to build a socioeconomic status (SES) index by transforming each one into a three-category and ordinal variable, and summing points for each individual to build a scale between 5 and 12 points. Low SES was assigned to a sum of 5–7 points, medium SES level to 8–9 points, and high SES to 10–12 points.

### Statistical methods

We performed statistical analysis with Stata version 8 (StataCorp., College Station, TX, USA). Pearson correlations and analysis of variance (ANOVA) between groups of demographic variables were performed to evaluate their association with UAs or the difference between different UAs concentration (50 μg/L cutoff). To evaluate the association of UAs and its metabolites with cognitive performance, we log-transformed the cognitive tests scores when required to fit a normal distribution. Three tests were omitted in the analysis: It was not feasible to analyze the data because variables couldn’t be normalized. Those that fitted a normal distribution were evaluated with linear regression models, and those that did not were evaluated with logistic regression at the cutoff points based on the median value. Models were adjusted for variables that were found to be significantly correlated with at least two of the cognitive tests scores at *p* < 0.05: children’s age, children’s sex, mother’s school education level, Hb concentration, and PbB. The interaction between UAs and PbB was also included in the models when it was significant at *p* < 0.10. The models adjusted the standard errors for clustering on children’s school to correct the intraschool correlation. These analysis were also performed stratified by subjects with UAs concentration ≤50 μg/L and > 50 μg/L. Because of the high influence of sex in UAs concentrations, the models were also stratified for boys and girls to investigate individual effects; in these analyses sex was removed from other adjusting variables. Collinearity diagnosis was run for all models to confirm the absence of multicollinearity within independent variables.

## Results

Demographic and biochemical characteristics of subjects are shown in Table 1. The mean ± SD of UAs was 58.1 ± 33.2 μg/L; 52% of the children had UAs concentrations > 50 μg/L, and 10% had UAs concentrations > 100 μg/L. Mean PbB concentration was 11.5 ± 6.3 μg/dL, and 50.7% of children had PbB above 10 μg/dL. The percentages of children with Hb, ferritin, and zinc deficiency were 9.8, 11.7, and 27.7%, respectively.

The association of sociodemographic variables with UAs is shown in Table 2. A significant difference was found between boys and girls in all As compounds: UAs was 11.85 μg/L higher in boys than in girls (*p* < 0.01). UAs concentration was also associated with SES: UAs in the low SES group was significantly higher than in medium and high SES groups (*p* < 0.01). Children of parents who had a high school or college education excreted less UAs than children of those who had primary or no education (*p* < 0.01). Children’s age was also associated with UAs concentration; younger children (6 years of age) excreted more UAs and MMAs (*p* < 0.01) and more InAs and DMAs (*p* < 0.05) than older children (7–8 years of age). PbB correlated positively with UAs concentration (Pearson *R* = 0.158, *p* < 0.01) (Tables 2 & 3). Nutritional status indicators were not related to UAs concentrations (Table 3).

The overall unadjusted cognitive scores stratified by UAs concentration levels are shown in Table 4. Children in the group with high UAs concentration presented lower scores in 7 of 11 cognitive tests than children with low UAs concentration.

Table 5 shows the covariate-adjusted relationship between UAs concentration and cognitive performance. The analysis is also presented separately by sex and by UAs below and above 50 μg/L. Overall, a significant inverse association was found between UAs and the Visual–Spatial Abilities with Figure Design, the PPVT, the WISC-RM Digit Span subscale, and the Visual Search and Letter Sequencing Tests (*p* < 0.05). In the analysis stratified by UAs concentration, UAs was significantly associated with the PPVT, the WISC-RM Digit Span Subscale, the Sternberg Memory Test, and the Visual Memory Span at UAs levels ≤50 μg/L. In children with UAs > 50 μg/L, the Visual–Spatial Abilities with Figure Design, the Stimulus Discrimination, and Letter Sequencing Tests were significant at *p* < 0.05. For boys, the Visual–Spatial Abilities with Figure Design and the PPVT, Visual Search, and Letter Sequencing Tests had an inverse association with UAs (*p* < 0.05). And for girls, there was a significant negative association of UAs only with the WISC-RM Digit Span Subscale (*p* < 0.05). The significant associations of each metabolite with the cognitive function tests are also shown in Table 5 (type of arsenic in parentheses). In general, organic forms of As in urine affected more cognitive tests than did the inorganic forms.

## Discussion

We found an association of UAs with several cognition tests such as Visual–Spatial Abilities with Figure Design, the PPVT, the WISC-RM Digit Span subscale, and the Visual Search and Letter Sequencing Tests. These associations were independent from sociodemographic variables, nutritional status, and PbB. When analysis was stratified by UAs, some tests were associated with UAs at concentrations < 50 μg/L. However, some tests were not associated with the high UAs group even though the association was significant in the low UAs group and in the total sample; this might be attributed to the power reduction and increased variability in the high UAs group compared with the variability in the low UAs group. Cognitive tests that showed association with UAs represent complex cognitive processes such as memory, problem solving, and attention. Previous studies had shown an adverse relationship between As exposure and IQ (Calderon et al. 1999) and neurobehavioral performance (Rodriguez et al. 2003). Results from our study confirm earlier findings by Calderón et al. (2001) and Tsai et al. (2003) relating As exposure to memory alterations. These associations occurring even in children without elevated As concentrations could be caused by several possible mechanisms. As crosses the blood–brain barrier and has a wide range of effects on the white matter in the brain (Osterberg and Kernohan 1934). Evidence also shows that arsenite inhibits the synthesis and liberation of acetylcholine in brain slices (Kobayashi et al. 1987), and increases the monoamine activity in rat nervous system (Mejía et al. 1997; Tripathi et al. 1997). The increase in one monoamine, 5-hydroxyindole-3 acetic acid, is potentially neurotoxic (Jones et al. 2005; Mejía et al. 1997). Whatever the mechanism, the present investigation shows that exposure to As is associated with deficits in cognitive performance among school-age children, even at low exposure levels, affecting complex cognitive processes such as memory and problem solving, which could potentially interfere with performance at school.

We found different associations between UAs and cognitive tests in boys and girls. Several cognitive tests were negatively associated with UAs only in boys, and the Digit Span subscale, an evaluation of memory, was significantly associated with UAs only in girls. InAs seemed to affect only boys in the Letter Sequencing Test. According to ANOVA to detect differences in cognitive performance between boys and girls (data not shown), differences in associations of cognitive tests with UAs between boys and girls did not seem to be related with differences in sex. Although it has also been reported by others (Chen et al. 2003; Kristiansen et al. 1997; Vahter et al. 2007), it is unclear why boys would excrete more UAs, which thereby affects their cognitive performance.

SES had an inverse association with UAs in our study. Ahmad et al. (2001) also found an inverse association between arsenicosis and SES and education level, probably due to the lack of access to As-free water. In other studies, As was also related to nutritional status of participants. Sikder et al. (2005) found that most of the arsenicosis patients in their study also suffered from malnutrition. Minamoto et al. (2005) reported that children living in households with As-contaminated tube well water had lower height for their age than children living in noncontaminated households. Similarly, Islam et al. (2004) found higher rates of underweight in individuals with arsenicosis than in controls, and concluded that poor nutritional status increased the complications of arsenicosis. In our study we did not find an association between UAs excretion and height, weight, and iron and other minerals’ concentration in serum. The lack of association was probably owing to the fact that our subjects were generally well nourished. Only 2% were stunted and 10% were anemic; and the rate of children with high concentration of UAs was the same among anemic and nonanemic children.

In conclusion, our study of a population of children living in an area contaminated with both As and lead showed that As contamination affected children’s cognitive function independent of any effect of lead, even in children with UAs below the safe declared concentration limit of 50 μg/L (ATSDR 2000).

## Figures and Tables

**Table 1:** Characteristics of study participants.

Variables	Value
No.	591
Percent male	54
Age (months)	83.4 ± 4.4
Socioeconomic level (%)	
Low	27.5
Medium	50.1
High	22.4
Mother’s school level (%)	
Primary or no education	25.6
Junior high school	55
High school or college	19.4
Weight for height: < 2 SD (%)	3
Weight for age: < 2 SD (%)	1.2
Height for age: < 2 SD (%)	2.2
Hb (g/dL)	13.4 ± 0.8
< 12.4 (%)	9.8
Ferritin (μg/L)	27.2 ± 16.1
< 12 (%)	11.7
ZPP (μmol/mol heme)	65.8 ± 22.2
≥70 (%)	29.3
Zinc (μg/dL)	80.1 ± 24.7
< 65 (%)	27.7
Copper (μg/dL)	106.8 ± 21.0
< 80 (%)	11
PbB concentration (μg/dL)	11.5 ± 6.3
≥10 (%)	50.7
InAs (ug/L)	8.7 ± 6.1
MMA (ug/L)	7.7 ± 5.2
DMA (ug/L)	41.7 ± 24.1
UAs (ug/L)	58.1 ± 33.2
UAs > 50 (%)	52.3
UAs > 100 (%)	9.8

Values are mean ± SD or percent.

**Table 2:** Urinary arsenic comparison among sociodemographic, hemoglobin, and PbB concentration groups [no. (mean ± SD)].

Variables	No.	InAs	MMA	DMA	UAs
Sex					
Male	319	9.4 ± 6.9^a^	8.5 ± 5.5^a^	45.6 ± 26.0^a^	63.5 ± 35.9^a^
Female	272	7.8 ± 4.9^b^	6.8 ± 4.8 b	37.1 ± 20.7 b	51.7 ± 28.5^b^
SES					
Low	154	10.3 ± 6.7^a^	9.3 ± 5.8^a^	48.6 ± 26.2^a^	68.1 ± 36.7^a^
Medium	280	8.1 ± 5.6 b	7.3 ± 5.0 b	39.7 ± 23.8 b	55.6 ± 32.4 b
High	125	8.2 ± 6.4 b	7.2 ± 4.7 b	37.9 ± 21.4 b	53.3 ± 30.2 b
Mother’s school level					
Primary or no education	146	9.5 ± 6.8^c^	8.5 ± 5.7^c^	45.6 ± 25.9^a^	63.7 ± 35.6^a^
Junior high school	314	8.7 ± 6.2	7.7 ± 5.1	41.8 ± 24.5	58.0 ± 33.8
High school or college	111	7.7 ± 4.8^d^	7.2 ± 4.7^d^	37.6 ± 20.8 b	52.5 ± 28.7 b
Age group (years)					
6	297	9.3 ± 6.8^c^	8.3 ± 5.4 a	44.2 ± 24.9^c^	61.7 ± 34.7^a^
7–8	294	8.0 ± 5.3^d^	7.2 ± 4.8^b^	39.2 ± 23.0^d^	54.3 ± 31.3 b
PbB concentration					
≤10 μg/dL	293	7.8 ± 5.1 a	7.1 ± 4.9 a	38.5 ± 22.1^a^	53.4 ± 30.3^a^
> 10 μg/dL	297	9.6 ± 6.9 b	8.4 ± 5.3 b	44.7 ± 25.6 b	62.6 ± 35.4 b

a,b Different letters represent significant differences among rows at *p* < 0.01.

c,d Different letters represent significant differences among rows at *p* < 0.05.

**Table 3:** Anthropometric and nutritional variables in children with high and low total urinary arsenic concentrations.

	UAs ≥ 50 μ g/L		UAs < 50 μg/L	
Variables	No.	Mean ± SD	No.	Mean ± SD
Height for age (*Z*-score)	308	−0.10 ± 0.92	281	−0.13 ± 1.02
ZPP (μmol/mol heme)	308	66.36 ± 24.99	282	65.09 ± 18.81
Hb (g/dL)	309	13.36 ± 0.81	282	13.35 ± 0.83
Ferritin (μg/dL)	290	27.31 ± 16.65	264	27.13 ± 15.55
Zinc (μg/dL)	299	78.79 ± 23.09	267	81.63 ± 26.27
Copper (μg/dL)	260	107.78 ± 20.88	239	105.75 ± 21.10
Weight (kg)	309	25.28 ± 5.47	282	24.78 ± 5.69
Height (cm)	309	120.89 ± 5.22	282	120.82 ± 5.81
Head circumference (cm)	309	50.65 ± 1.47	282	50.54 ± 1.38
Knee height (cm)	309	36.86 ± 2.05	282	36.82 ± 2.19

No significant differences were found between groups (one-way ANOVA).

**Table 4:** Cognitive scores of children stratified by UAs concentration [mean ± SD (minimum–maximum)].

Cognitive tests	Overall	Children with UAs < 50 μg/L	Children with UAs > 50 μg/L
Math Achievement Test	31.35 ± 7.50 (3–52)	32.27 ± 7.69 (8–52)	30.57 ± 7.20 (3–49)*
Visual–Spatial Abilities with Figure Design	18.31 ± 5.15 (2–34)	18.88 ± 5.16 (3–34)	17.84 ± 5.08 (2–31)*
WISC-RM Arithmetic Subscale	7.41 ± 3.62 (1–17)	7.26 ± 3.66 (1–17)	7.59 ± 3.57 (1–17)
Peabody Picture Vocabulary Test	103.19 ± 15.65 (55–145)	105.20 ± 16.11 (55–145)	101.67 ± 14.92 (55–140)*
WISC-RM Digit Span Subscale	9.10 ± 3.63 (1–19)	9.46 ± 3.73 (1–19)	8.80 ± 3.55 (2–18)*
Sternberg Memory (correct trials)	12.14 ± 2.94 (4–20)	12.30 ± 3.01 (4–20)	11.98 ± 2.86 (5–20)
Visual Memory Span (correct trials)	2 ± 0.52 (0.69–3.37)	2.03 ± 0.51 (0.69–3.37)	1.97 ± 0.53 (0.69–3.26)
Stimulus Discrimination (correct trials < 19 vs. ≥19)	0.57 ± 0.50 (0–1)	0.63 ± 0.48 (0–1)	0.52 ± 0.50 (0–1)*
WISC-RM Coding Subscale	2.26 ± 0.59 (1–3.71)	2.29 ± 0.58 (1.07–3.71)	2.23 ± 0.61 (1–3.71)
Visual Search (correct minus incorrect minus omitted trials)	5.03 ± 1.51 (1–10.82)	5.23 ± 1.47 (1.14–10.82)	4.84 ± 1.50 (1–8.99)*
Letter Sequencing (correct trials 0 vs. ≥1)	0.48 ± 0.50 (0–1)	0.55 ± 0.50 (0–1)	0.41 ± 0.49 (0–1)*

* Difference between children with UAs < 50 and children UAs > 50 μg/L is significant at *p* < 0.05.

**Table 5:** Covariate-adjusted association between UAs (μg/dL) and tests of cognitive function.

Cognitive test	Overall (*n* = 557)	Children with UAs < 50 μg/L(*n* = 267)	Children with UAs > 50 μg/L(*n* = 290)	Males (*n* = 306)	Females (*n* = 251)
Linear regressions [beta coefficient (95% CI)]					
Problem Solving and Vocabulary					
Math Achievement	−0.023 (−0.052 to 0.006) (MMA*)	−0.098* (−0.199 to 0.003) (DMA,* MMA*)	−0.015 (−0.047 to 0.018)	−0.022* (−0.046 to 0.001) (DMA,* MMA#)	−0.019 (−0.063 to 0.026)
Visual–Spatial Abilities with Figure Design	−0.024** (−0.045 to −0.004) (DMA**)	−0.018 (−0.096 to 0.061)	−0.028** (−0.053 to −0.004) (DMA**)	−0.026** (−0.048 to −0.003) (DMA*)	−0.019 (−0.045 to 0.007)
WISC-RM Arithmetic Subscale	−0.001 (−0.016 to 0.014)	−0.014 (−0.051 to 0.024)	−0.006 (−0.029 to 0.016)	−0.000 (−0.020 to 0.020)	−0.003 (−0.021 to 0.015)
Peabody Picture Vocabulary Test	−0.064** (−0.115 to −0.013) (InAs,** DMA,** MMA*)	−0.185# (−0.293 to −0.078) (DMA,** MMA*)	−0.058* (−0.120 to 0.004) (DMA*)	−0.058** (−0.105 to −0.010) (DMA,** MMA**)	−0.070 (−0.169 to 0.029)
Memory					
WISC-RM Digit Span Subscale	−0.014** (−0.025 to −0.002) (DMA,* MMA**)	−0.037** (−0.065 to −0.010) (DMA**)	−0.012 (−0.037 to 0.012)	−0.008 (−0.025 to 0.008)	−0.039# (−0.062 to −0.016) (DMA,# PbB ×UAs**)
Sternberg Memory(correct trials)	−0.002 (−0.007 to 0.004)	−0.027** (−0.053 to −0.002) (DMA*)	0.002 (−0.008 to 0.012)	0.002 (−0.006 to 0.010)	−0.008 (−0.020 to 0.005)
Visual Memory Span(correct trials)^a^	−0.000 (−0.002 to 0.001)	−0.003** (−0.007 to 0.000) (DMA**)	−0.001 (−0.002 to 0.003)	0.000 (−0.002 to 0.002)	−0.001 (−0.004 to 0.002)
Attention					
WISC-RM Coding Subscale^a^	0.000 (−0.001 to 0.000) (DMA*)	0.000 (−0.005 to 0.005)	−0.001 (−0.003 to 0.002)	0.000 (−0.001 to 0.002)	−0.002 (−0.004 to 0.001)
Visual Search (correct minus incorrect minus omitted trials)^a^	−0.007# (−0.011 to −0.002) (InAs,# DMA,** MMA**)	−0.008 (−0.022 to 0.005)	−0.006* (−0.012 to 0.000) (DMA**)	−0.016** (−0.029 to −0.003) (DMA,# MMA,** PbB ×UAs*)	0.010* (0.000 to 0.019) (PbB ×UAs#)
Logistic regressions [odds ratio (95% CI)]					
Memory					
Stimulus Discrimination(correct trials < 19 vs. ≥19)	0.998 (0.993 to 1.004)	0.982 (0.957 to 1.008)	1.004** (1.000 to 1.008) (DMA*)	1.002 (0.995 to 1.009)	0.990 (0.977 to 1.002)
Attention					
Letter Sequencing(correct trials 0 vs. ≥1)	0.992# (0.987 to 0.996) (DMA,# MMA#)	0.992 (0.963 to 1.021)	0.993** (0.988 to 0.999) (DMA#)	0.988# (0.982 to 0.993) (InAs,** DMA,# MMA#)	0.998 (0.990 to 1.007)

Values are beta coefficient [95% confidence interval (CI)] of total urinary arsenic from a multiple regression, or odds ratio from a logistic regression, with cognitive test score as the dependent variable. Regression models were adjusted for age in months, sex, mother’s school level, Hb concentration, PbB concentration, and for PbB concentration ×. UAs interaction when it was significant at *p* < 0.10. CIs were adjusted for clustering on children’s school. Significant effect of UAs metabolites on cognition functions or PbB concentration × UAs interaction is shown in parentheses.

a These cognitive tests were log-transformed to achieve the normal distribution. Beta coefficient significant at

* *p* < 0.1,

** *p* < 0.05,

# *p* < 0.01.
